# Calves’ management conditions affect sperm count in adult bulls

**DOI:** 10.1186/s13028-020-00563-x

**Published:** 2020-11-25

**Authors:** Tomaž Snoj, Kaja Blažič, Nika Šehić, Tilen Vake, Gregor Majdič

**Affiliations:** Veterinary Faculty, University of Ljubljana, Gerbičeva 60, 1000 Ljubljana, Slovenia

**Keywords:** Artificial insemination, Bull, Maternal separation, Semen parameter, Sire

## Abstract

**Background:**

Early maternal separation may have long-lasting physiological effects on different organ systems. Although long-lasting effects of early maternal separation are mostly studied in connection with the development and function of the central nervous system hypothalamic–pituitary–adrenal axis, animal health, behaviour and productivity, there is a lack of information about its impacts on the reproductive system. In the dairy industry, calves are often separated from mothers 1 or 2 days after birth and are not nursed. In the present retrospective study based on data from an artificial insemination (AI) centre, we compared semen parameters and fertility in bulls that were separated from their mothers one day after birth with the semen parameters of bulls that remained with their mothers and were nursed for approximately 2 months. Semen parameters were followed in 3 consecutive years in 52 maternally separated and 22 nursed bulls.

**Results:**

Ejaculate volume and total sperm count in ejaculate were significantly higher in nursed bulls in comparison to maternally separated bulls at the age 25–36 and 37–48 months, but interestingly, not at the age 12–24 months, during the first year in the AI centre. Non-return rates did not differ between separated and nursed bulls.

**Conclusion:**

The results suggest that early maternal separation causes long-lasting effects on the functioning of the male reproductive system, evident by reduced production of semen in adult bulls. The data suggest that with a standard of 20 million sperms per straw of frozen semen, 27–78 fewer straws can be obtained from one ejaculate of maternally separated bulls in comparison to the nursed bulls.

## Background

At many dairy farms, separation of calves from cows immediately after birth is a common practice. Instead of suckling with their mothers, calves are fed with milk or milk substitute by milk feeder until they begin to consume hay or silage.

Many studies in rats and mice have shown that early neonatal maternal separation is associated with altered behaviour and neuroendocrine responses in adulthood. Early maternal separation causes postnatal stress and permanently alters the hypothalamic–pituitary–adrenal (HPA) axis, resulting in higher basal plasma ACTH levels and a blunted response to stress in the adult animal [1]. It also increases anxiety-like behaviour, inter-male aggression, and affects social behaviour and memory function in both rats and mice [2–5]. Although the influence of early maternal separation on later behaviour and HPA axis activity is well established, its effects on the reproductive functions is less clear. Lau et al. [6] studied the onset of puberty, sperm count, regularity of the estrus cycle, weight of reproductive organs, and the number of pups in rats exposed to maternal separation during neonatal life. There were no significant differences between groups in female rats, while in males, testes weights were reduced by 8%, but no other reproductive parameters were affected. Several other studies also did not report any effects of neonatal maternal separation on reproductive parameters and male sexual behaviour in adult males [7, 8]. However, a study by Bodensteiner et al. [9] reported delayed puberty and lower testosterone levels in maternally separated adult male rats, although sperm count did not differ between maternally separated and control groups. Reduced blood testosterone levels were also determined in prenatally stressed adult male mice [10]. Furthermore, some studies also reported the influence of maternal separation on sexual behaviour. Maternal separation for 6 h daily from postnatal days 2 to 10 caused differences in male sexual behaviour such as increased latency to mount and intromit, and reduced percent of ejaculating males [11]. Similarly, Bodensteiner et al. [9] reported reduced male sexual behaviours in maternally separated male rats.

Studies addressing the impact of maternal separation on adult cattle have mainly focused on health, welfare, behaviour, and productivity [12, 13]. A study by Wagner et al. [14] reported that adult cows that were maternally separated when neonate had higher basal cortisol levels in comparison to adult cows that nursed when young. During the isolation stress test, nursed cows explored more squares in the arena than maternally separated cows, suggesting increased fear/anxiety in maternally separated cows. As suggested by Roth et al. [15], maternal deprivation stress, together with increased cortisol secretion, could be associated with lower weight gain, as maternally separated animals consume less feed and have a higher incidence of health problems such as diarrhoea. Cow-calf contact during the first weeks of life has several beneficial effects including higher weight gain, lower incidence of abnormal behaviours such as tongue-rolling and cross-suckling, and lower incidence of gastrointestinal disorders in calves [16]. Although the impact of maternal separation on health, behaviour, and productivity has been studied, there is a lack of information about the impact of early maternal separation on reproductive parameters.

In the present study, we compared semen quality parameters of bulls that were nursed by their mothers as calves or were maternally separated a few days after birth. We hypothesized that if early postnatal stress caused by maternal separation permanently or transiently affects the HPG axis, this will be reflected in the bulls’ semen quality.

## Methods

### Animals and data collection

Data on semen quality were collected at the artificial insemination (AI) centre in Preska, Slovenia. The owner of the bulls (AI Centre Preska) provided the data and gave us consent to use the data freely for research purposes.

Since this was a retrospective study using data obtained during regular procedures with bulls in the AI centre, no animal experiments were performed for the purpose of this study and therefore, no ethical approval was needed.

In total, 74 Brown Swiss bulls were included in this study, of which 52 bulls were separated from their mothers one day after birth and fed with milk or milk substitute using milk feeders. Twenty-two bulls remained with their mothers and were nursed for two months. All bulls were relocated from the home farm to the rearing facility for young bulls. The age of bulls at the relocation was 84–153 days.

At the age of one year (± 10 days), the scrotal circumference was measured by measuring tape at the scrotum’s widest point.

At the age of 12—14 months, bulls were relocated to the AI centre. During their stay they were tethered in individual boxes. They were fed 9–10 kg of hay and 1.5 kg of complementary feed twice per day and had drinking water ad libitum. Bulls were provided with all the necessary veterinary care. All bulls were subjected to the same standard mounting procedure with the same intervals (3–4 days) between two semen collections. In some rare cases, the interval between two ejaculations in some bulls was longer because of health issues. During semen collection, bulls were mounting a teaser animal. One or two ejaculates on a single day were collected using an artificial vagina. In this study, data of semen parameters during the first and second ejaculations were analysed. In total, data from 4085 ejaculates from the 52 maternally separated bulls and 1210 ejaculates from the 22 nursed bulls were included. The semen parameters were further divided by bulls’ age into three groups: 12–24, 25–36, and 37–48 months of age.

Ejaculate volume was determined directly from the graded collection tubes. Sperm concentration in mL of semen was evaluated by a photometer (IMV Technologies, L’Aigle, France). Total sperm output was calculated by multiplying ejaculate volume and sperm concentration.

Further, the fertility of both groups of bulls was estimated by a statistical comparison of the non-return rate (NR (%)) for a period of 90 days.

### Statistical analyses

All data for semen characteristics were analysed using repeated measures ANOVA with the age and management conditions (nursing or maternal separation) conditions as independent variables, an individual bull as a subject variable, and consecutive semen collection (the first or second ejaculation) as within factor. ANOVA was followed by a *post-hoc* Tukey–Kramer test. Data for the scrotal circumference and NR were analysed by one-way ANOVA, followed by *post-hoc* Tukey–Kramer test. Results are shown as mean ± SE. Pearson correlation coefficient was calculated to determine the correlation between scrotal circumference at 12 months of age and average semen volume, sperm count, and sperm concentration during the age of 12 to 24 months. Differences were considered statistically significant with P < 0.05.

## Results

### Scrotal circumference

Scrotal circumference at 12 months of age was 35.5 ± 0.3 cm in maternally separated bulls and 36.5 ± 0.4 cm in nursed bulls. The values did not differ significantly. There was no correlation between scrotal circumference and average ejaculate volume, sperm count, and sperm concentration with *R*^2^ 0.05, 0.137, and 0.008, respectively.

### Semen parameters

**Fig. 1:**
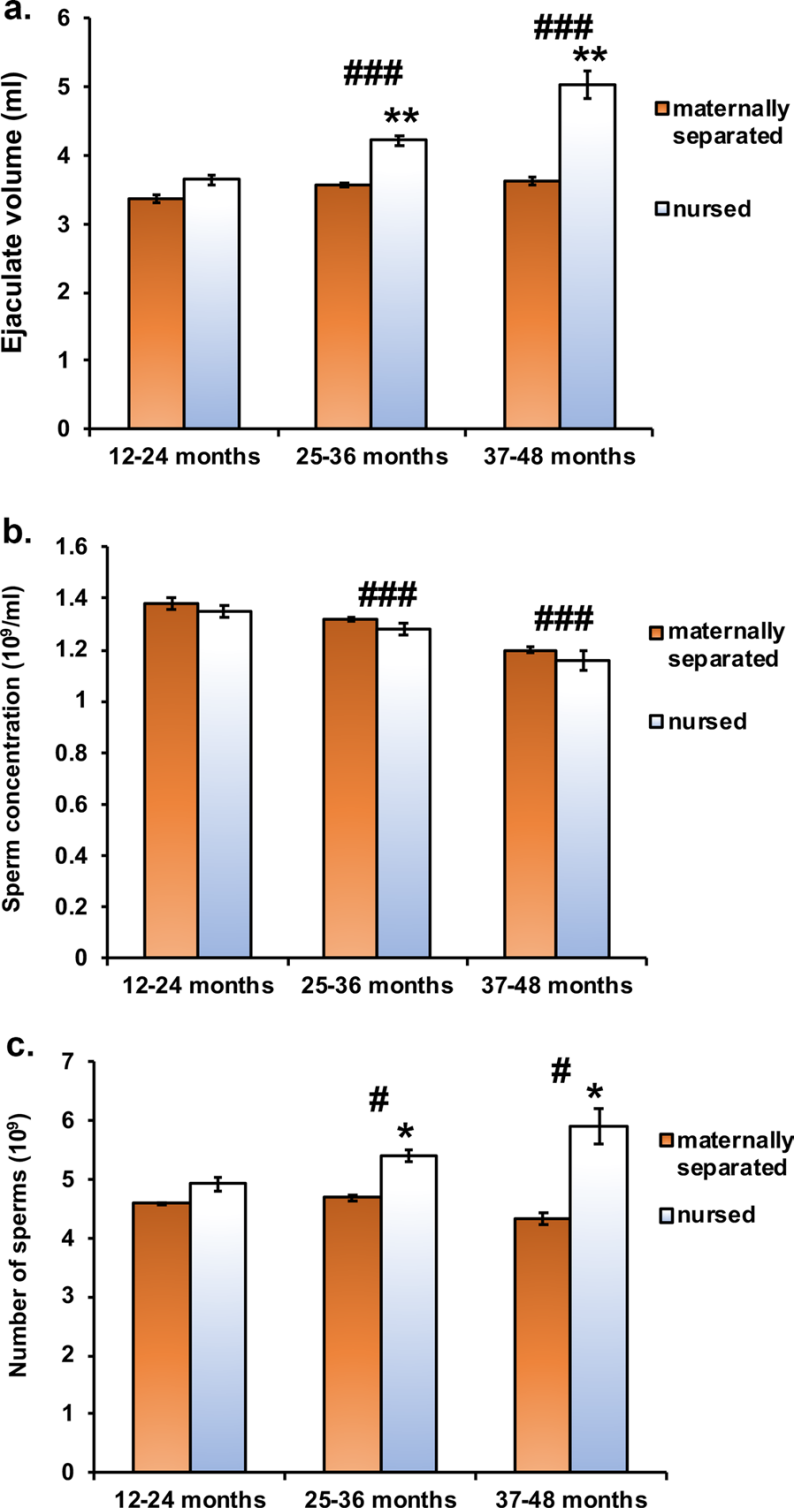
Both ejaculate volume (**a**) and total sperm count (**c**) differed between groups with regards to the management conditions and age (**P < 0.01 (management conditions); ###P < 0.001 (age) for ejaculate volume and *P < 0.05 (management conditions) and #P < 0.05 (age) for total sperm counts). In both parameters the difference was present in 3 and 4 years old bulls but not in 2 years old bulls with nursed bulls having larger ejaculate volume and higher total number of sperms in comparison to maternally separated bulls. Concentration of sperms in ejaculate (**b**) did not differ with regard to breeding conditions, but was significantly different with regard to the age (###P < 0.001)

Repeated measures ANOVA analyses of semen parameters revealed that nursed bulls had a significantly higher total volume of collected semen in comparison to maternally separated bulls (Fig. 1) with a significant effect of neonatal separation (P < 0.01), age (P < 0.001), and interaction between neonatal separation and age (P < 0.001). The *post-hoc* test revealed no difference in semen volume in 12–24 months old bulls, but there was a significant difference between nursed and maternally separated bulls at 25–36 and 37–48 months of age.

Similarly, total sperm count in ejaculate was different between groups with a significant effect of management conditions (P < 0.05), age (P < 0.05), and interaction between management conditions and age (P < 0.05). The *post-hoc* test revealed a significant difference between nursed and maternally separated bulls in total sperm count in 25–36 and 37–48 months old bulls, but not at 12–24 months of age.

The concentration of sperms did not differ between nursed and maternally separated bulls (Fig. 1). However, there was a significant effect of age (P < 0.001). The *post-hoc* test revealed that concentration was significantly different in each age group regardless of management conditions.

### Non-return rate

The mean NRs (%) for maternally separated and nursed bulls were 71.6 ± 1.47 and 72.6 ± 2.04%, respectively. The values did not differ significantly.

## Discussion

We evaluated whether management conditions affected ejaculate volume, sperm concentration, total sperm output, and motility and fertility expressed as NR in Brown Swiss bulls. All bulls were from an AI centre and were as calves separated from mothers on the first day after birth or were nursed for 2 months.

Many studies have shown that neonatal stress has long-lasting consequences for the development of the brain and neuroendocrine system. Although the vast majority of these studies were performed in laboratory rodents, some studies also showed the effects of maternal separation in other animals such as pigs [17, 18] and also in humans. However, although we presume that maternal separation caused considerable stress to calves in our study, other factors could influence growth and development, such as differences in feeding (milk versus milk supplement).

We observed highly significant differences between nursed and maternally separated bulls in the ejaculate volume and in the total sperm count in the ejaculate, but not in the concentration of sperms. Interestingly, these differences were observed only in 25–36 and 37–48 months old bulls, but not in 12–24 months old nursed and maternally separated bulls. These results suggest that maternal separation influences gonadal development and sperm production, although, at present, it is not known how and through which mechanisms. As this was a retrospective study, we could not obtain blood samples to determine the levels of sex hormones. Therefore, further studies will be needed to examine whether neonatal stress perhaps permanently alters levels of hormones such as testosterone and follicle stimulating hormone (FSH), which are known to regulate spermatogenesis.

Wagner et al. [14] reported that maternally separated cows have higher basal cortisol levels in adulthood than non-separated. Similar data are not available for bulls, but if the response to maternal separation in bulls is similar to that in cows, high basal cortisol levels could reduce testosterone secretion from the testes [19, 20]. This could result in decreased sperm production and lower sperm count in adult bulls. However, nutrition during early life could also influence reproductive parameters. Enhanced nutrition plan during the first weeks of life results in increased testes’ size and seminiferous tubule diameter, together with an increased number of Sertoli cells and mature sperms cells in adult bulls. In the same study, smaller testes were detected in bulls subjected to a restrictive diet with growth limited to 0.5 kg per day [21]. In our study, we have not estimated the differences in nutrition between nursed and maternally separated bulls. However, bulls from both groups had access to feed, either from mothers or from milk feeders. It cannot be ruled out that differences in feed composition (milk or milk substitute) caused differences in the development of testes, what could later result in differences in sperm counts. So, besides stress due to maternal separation, differences in nutrition cannot be ruled out as a cause of differences in sperm parameters.

Scrotal circumference is usually linked to sperm production capacity. Several studies reported a significant correlation between scrotal circumference and semen parameters with ejaculate volume and total sperm output closely related to the testes size [22, 23]. In our study, data for scrotal circumference was available only for bulls at one year of age. Maternally separated bulls did not have significantly lower scrotal circumference at this age. Interestingly, though, sperm count at the initial year of the study was also not significantly different between groups, although bulls from the maternally separated group had lower sperm count. This is, therefore, in agreement with the data about the connection between scrotal circumference and sperm production capacity, although statistical analysis in our study did not reveal a correlation between scrotal circumference and semen parameters. However, we had measurements for scrotal circumference only at 12 months of age when semen collection just started, so it is plausible to assume that scrotal circumference changed with age. This is a likely reason why we did not find a correlation between these parameters. Unfortunately, data about scrotal circumference in older bulls were not available. Therefore, we could not correlate scrotal circumference and semen parameters at the age of 25–36 and 37–48 months, where significant differences would be expected, judging from the differences in total sperm count. Although the bulls included belong to the same breed, some individual genetic characteristics might influence the semen production capacity and fertility of both male and female progeny. Selection can have an important impact on testis size and semen production. Strong selection pressure could, therefore, cause individual differences in reproductive parameters on different farms, but these would be difficult to separate from the management practice. Undoubtedly, the interactions between selection, genetic drift, and management conditions are a very important and interesting area of research, although this is beyond the scope of our study.

Intriguing is the observation that differences in semen parameters were significant in bulls from 25–48 months of age, but not in 12–24 months old bulls. Bulls normally enter puberty around 9–10 months of age when they can produce their first ejaculates, but their reproductive tract continues to develop, and the capacity for sperm production increase for an extended period after the beginning of puberty. Scrotal circumference increases at least until 30 months of age, while sperm production (total sperm output) increases up to 5 years of age [24, 25]. In the present study, ejaculate volume and total sperm count increased during the observed period (12–48 months of age). However, the increase was more substantial in nursed bulls than in maternally separated bulls. This suggests that perhaps the final maturation of the testes and reproductive tract concludes earlier in maternally separated, presumably stressed bulls. Again, this could be due to increased basal cortisol levels, as cortisol is known to negatively influence the reproductive system’s function [19, 20]. A recent study in humans has shown that chronic stress could contribute to earlier puberty [26], leading to earlier sexual maturation. If similar effects are present in bulls, this could explain why differences in semen parameters in our study become prominent after 25 months of age.

NR rate shows the percent of successful first inseminations with the semen of a particular bull. Although NR is influenced by several factors such as AI service, heat detection, and cows’ health, it is an important parameter for evaluating bulls’ fertility. Statistical analysis showed no significant differences in NRs between maternally separated and nursed bulls. Therefore, these results suggest that rearing conditions did not affect the fertilizing ability of sperms, yet it reduced the production of sperms.

Although the bulls’ fertility as judged from the NR rate was not affected by neonatal rearing conditions, the results have important implications for the bovine AI industry. Reduced sperm count in maternally separated bulls does not affect their fertility in natural service. However, a significant difference in total sperm count per ejaculate does reduce the total number of insemination doses obtained from one ejaculate. Our results suggest that with a standard of 20 million sperms per straw of frozen semen, 27–78 fewer straws can be obtained from one ejaculate of maternally separated bulls in comparison to the nursed bulls. Besides the economic impact for semen collection organizations, this also means that maternally separated bulls will produce a lower number of calves what is important for cattle farming if such bulls have otherwise excellent progeny. Therefore, the AI organizations should pay attention to bulls’ neonatal rearing conditions, as neonatal stress could likely result in large and significant differences in total sperm output. We suggest that newborn calves, which will be included in the semen production, should not be separated from their mothers immediately after birth.

Further studies will be needed to establish how these effects of early life stress influence the development of the male reproductive tract, but the results clearly show the importance of the neonatal environment for the functioning of male reproductive organs.

## Conclusions

Early separation of male calves from their mothers influences sperm output in adulthood. Ejaculate volume and total sperm count in ejaculates of nursed bulls were higher in comparison to maternally separated bulls at 25–36 and 37–48 months of age, but not 12–24 months old bulls. Results suggest that early maternal separation causes long-lasting effects on the HPG axis, evidenced by reduced production of the semen in adult bulls. However, further prospective studies will be needed to confirm these results and explore the mechanisms leading to the reduced sperm counts in maternally separated bulls. Besides stress, other factors such as the difference in the nutrients in milk and milk substitute could potentially cause such differences. Nevertheless, the results of this study are important for the insemination industry since 27–78 fewer produced straws can be expected per ejaculate of maternally separated bulls in comparison to nursed ones.

## Data Availability

The datasets used and analysed during the current study are available from the corresponding author on reasonable request.
